# Exploiting RNA thermometer-driven molecular bioprocess control as a concept for heterologous rhamnolipid production

**DOI:** 10.1038/s41598-021-94400-4

**Published:** 2021-07-20

**Authors:** Philipp Noll, Chantal Treinen, Sven Müller, Lars Lilge, Rudolf Hausmann, Marius Henkel

**Affiliations:** Institute of Food Science and Biotechnology, Department of Bioprocess Engineering (150K), University of Hohenheim, Fruwirthstr. 12, 70599 Stuttgart, Germany

**Keywords:** Biotechnology, Industrial microbiology

## Abstract

A key challenge to advance the efficiency of bioprocesses is the uncoupling of biomass from product formation, as biomass represents a by-product that is in most cases difficult to recycle efficiently. Using the example of rhamnolipid biosurfactants, a temperature-sensitive heterologous production system under translation control of a fourU RNA thermometer from *Salmonella* was established to allow separating phases of preferred growth from product formation. Rhamnolipids as bulk chemicals represent a model system for future processes of industrial biotechnology and are therefore tied to the efficiency requirements in competition with the chemical industry. Experimental data confirms function of the RNA thermometer and suggests a major effect of temperature on specific rhamnolipid production rates with an increase of the average production rate by a factor of 11 between 25 and 38 °C, while the major part of this increase is attributable to the regulatory effect of the RNA thermometer rather than an unspecific overall increase in bacterial metabolism. The production capacity of the developed temperature sensitive-system was evaluated in a simple batch process driven by a temperature switch. Product formation was evaluated by efficiency parameters and yields, confirming increased product formation rates and product-per-biomass yields compared to a high titer heterologous rhamnolipid production process from literature.

## Introduction

A major part of a future bioeconomy-driven industrial biotechnology is based on sustainable bulk products with typically low added-value. Formerly mainly focused on products that are difficult to synthesize by chemical means, such as proteins or chiral molecules etc., industrial biotechnology will include typical low-molecular weight products that were exclusively produced by chemical means. As such, the efficiency of substrate conversion is of particular importance, as future processes of industrial biotechnology are therefore tied to efficiency requirements in competition with the chemical industry. A key challenge to advance the efficiency of bioprocesses is the uncoupling of biomass from product formation, as biomass represents a by-product that is in most cases difficult to recycle efficiently.

### Rhamnolipids

Microbial rhamnolipid biosurfactants (RL) as bulk chemicals represent a model system for future industrial biotechnology in the context of a bio-based economy. Biosurfactants are based on renewable substrates, whereas many conventional surfactants are derived from petrochemical sources^1^. Biosurfactants exhibit beneficial properties like low toxicity, biodegradability, high surfactant efficiencies and high stability at extreme pH, temperature and salinity when compared to many chemical surfactants^2^. The fields of application for surfactants range from the cosmetic sector (personal care products), the food sector (emulsifiers or stabilizers) and the pharmaceutical sector (antimicrobial and antifungal activities) to enhanced oil recovery and cleaning agents (household detergents)^2–6^. Unsurprisingly, an increase in research interest on microbial biosurfactants could be observed over the past decade^7^. However, production of biosurfactants is still cost intensive, especially the downstream processing^8^. Furthermore, the use of the model organism *P. aeruginosa*, even though it is reported to produce the highest RL titers (39 g/L)^9^, bares two disadvantages for a commercial production of the biosurfactant RL. First, *P. aeruginosa* is a human pathogen^10^ and second, RL production is controlled by a complex regulatory cell density-dependent mechanism called quorum sensing. To overcome these obstacles, heterologous RL production was first described by *Pseudomonas putida*^11, 12^. In 2016, the *rhlAB* genes were transferred from *P. aeruginosa* with the pBBR1MCS-3 based plasmid pSynPro8oT_*rhlAB* into the nonpathogenic host *P. putida* KT2440 resulting in heterologous constitutive expression of rhamnosyltransferase genes needed for mono-RL production^13^. A maximum concentration of 14.9 g/L RL could be reached in a fed batch process with a two-phase glucose feeding profile. However, this approach for heterologous RL production suffers from considerable biomass production which represents a major carbon sink (roughly 50% more biomass than product)^14^. This challenge is addressed with the presented study.

### RNA thermometer

Temperature fluctuations can be detected by different molecular thermosensors made of DNA, RNA, proteins or lipids. The different principles of temperature sensing have been extensively reviewed^15–17^. The thermosensors of interest for this study are molecular structures mostly found in the 5'-UTR of the mRNA that enable thermosensing. Due to the physical properties of RNA, so-called hairpin structures can be formed. In these hairpin structures, base pairing blocks the Shine-Dalgarno sequence, and the AUG start codon, which are part of the ribosomal binding and translation initiation site. A certain amount of thermal energy, depending on the composition of base pairs, is required to melt the H-bridges in the hairpin structure. By raising the temperature, the hairpin structure gradually opens and allows ribosomes to access the translation initiation site^16^. In 1989, the first *cis* encoded regulatory RNA element determining temperature-dependent translation initiation was discovered in the *cIII* gene of bacteriophage λ^18^. Up until today several temperature-sensitive RNA structures, governing mainly the expression of heat shock and virulence genes have been reported^19–23^ but to our knowledge not leveraged for industrial applications. This study aims to provide a proof-of-concept process exploiting a temperature responsive RNA thermometer (RNAT) for bioprocess control.

### ROSE-like RNA thermometer

In *P. aeruginosa* RL production is governed by an RNA-based temperature-sensing element an RNAT. RNATs are instrumental in an adequate response to the decisive signal of a temperature-shift from low environmental to elevated temperatures e.g. of 37 °C in a host system. The consequence after successful invasion of a host by a pathogen is the upregulation of its virulence factor production. To respond to elevated host temperatures, the pathogen *P. aeruginosa*, has a so termed ROSE (Repression Of heat-Shock gene Expression)-like RNAT. The RNAT is located in the 5’ UTR (untranslated region) of the *rhlA* mRNA, thermo-regulates the translation of the mRNA and therefore indirectly the production of the virulence factor RL. Almost all known ROSE-like RNATs govern heat shock gene expression with the exception of the ROSE-like element found to control the formation of the virulence factor RL in *P. aeruginosa* and in the heterologous system *P. putida* KT2440 pSynPro8oT_*rhlAB*^24, 25^. It was argued that at non-permissive environmental temperatures (≤ 30 °C) translation of *rhlA* is inhibited by the RNAT, which in turn interferes with the transcription of the *rhlB* gene. This phenomenon is known as polar effect^24^. In 2019, a ROSE-like RNAT structure was evaluated for temperature-responsivity and its ability to govern a temperature dependent expression of the *rhlAB* genes^25^. The latter study showed the potential to use RNATs as regulatory elements; however, the evaluated ROSE-like RNAT showed a limited regulatory effect on RL production. Consequently, a different temperature responsive element with a more pronounced regulatory effect on translation was investigated in this study.

### FourU RNA thermometer

**Figure 1:**
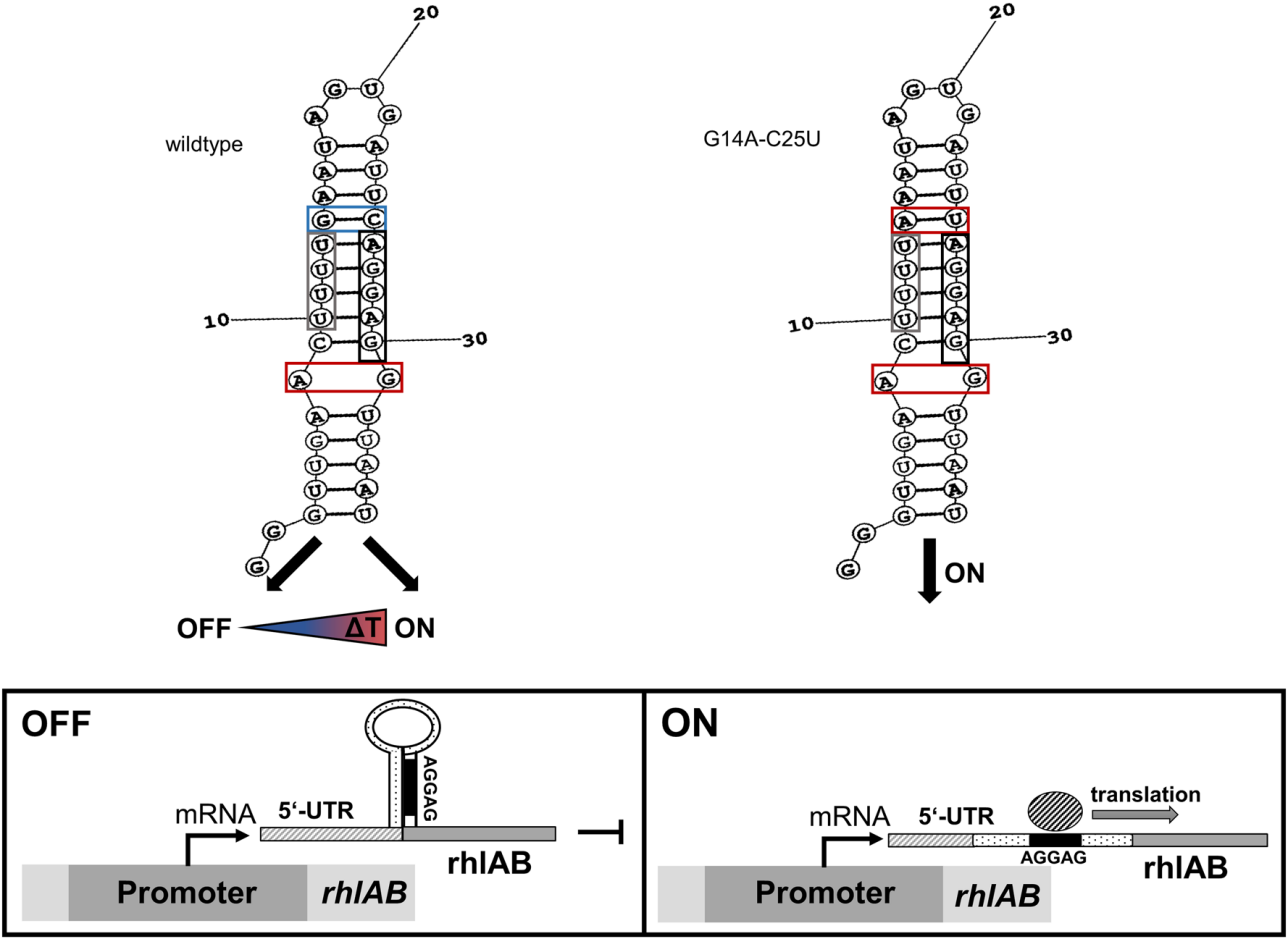
Working principal, hairpin structure and RNA sequence of *Salmonella* wild type fourU RNAT and destabilized (G14A-C25U) mutant used in this study. Base pairs framed in red are less stable than base pairs framed in blue, grey frames indicate the fourU motive and black frames the Shine-Dalgarno sequence. (Adapted from^29^).

Another family of RNATs are the fourU-type RNATs. FourU RNATs located mostly in the 5’UTR, are between 40 and > 270 nucleotides in length (*htrA*; *E. coli* and *shuA*; *Shigella dysenteriae*) and consist of 1–5 hairpin structures^26–28^. The temperature responsive “hairpin 2” of the 57 nucleotides *Salmonella* fourU-type RNAT was used in this study. In *Salmonella enterica* the fourU RNAT governs the expression of the heat shock gene *agsA*. In vivo experiments showed that the temperature responsive wild type fourU RNAT mostly prevents translation of a subsequent *bgaB* gene at 30 °C but is upregulated at common mammalian host temperatures of 37 °C and above. Previous studies reported that a destabilized variant of the wild type fourU RNAT can be obtained by introducing a single point mutation into the hairpin structure. This structure is destabilized at the temperature range investigated here allowing for an earlier onset of translation initiation (at lower temperatures) than the wild type RNAT (Fig. 1)^29^.

### Aim of this study

The presented study was meant to be a case study on the applicability of RNAT in combination with temperature changes to govern an exemplary bulk chemical production process within the framework of an industrial biotechnology, in this case microbial RLs. A temperature-sensitive heterologous RL production system under translation control of a fourU RNAT from *Salmonella*^29^ (Fig. 1) was established. The characteristics of this system are assessed in comparison to a destabilized structure used as a control that allows translation already at lower temperatures. The use of RNATs for molecular control through partial decoupling of biomass and product formation in bioprocesses is illustrated using RL biosurfactants as an example and presented by growth respectively product formation rates and yields. Furthermore, a proof-of-concept of a bioreactor cultivation with a temperature switch was carried out to elucidate the production capacity and potential of the molecular temperature switch for bioprocess control. Hence, this work marks a fundamental step for temperature-based (molecular) process design and control by exploiting temperature responsive elements.

## Results

To demonstrate the effect of temperature on RL production and cell growth, a set of experiments, comparing shake flask cultivations at different temperatures (20—42.5 °C), was conducted.

### Heterologous fourU RNA thermometer thermo-regulates RL production

**Figure 2:**
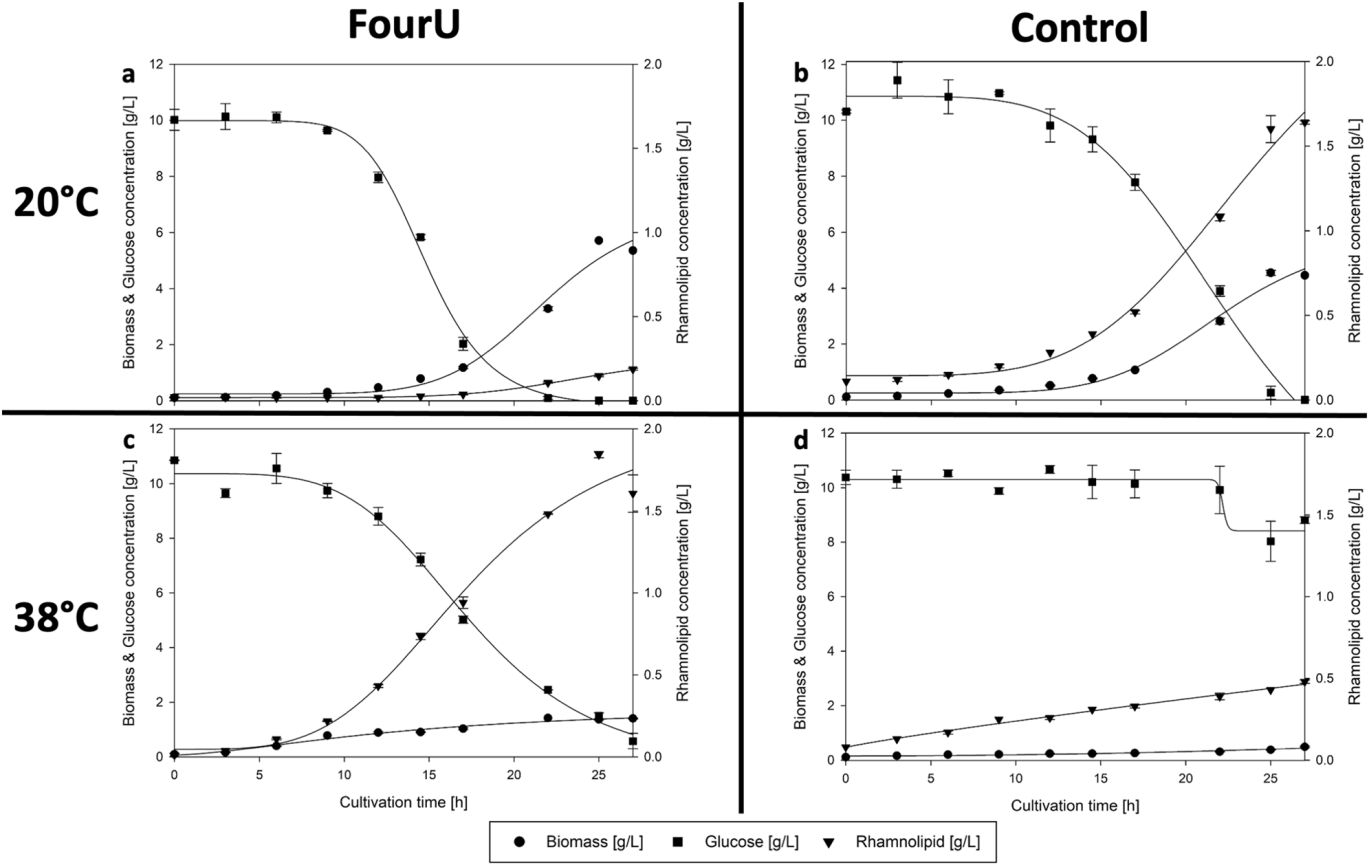
Time course of biomass- (circles), rhamnolipid- (triangles) and glucose concentration (squares) during shake flask cultivation of *P. putida* KT2440 pSynPro8oT_4U_*rhlAB* and pSynPro8oT_4U(G14A-C25U)_*rhlAB* control strain on ModR medium with 10 g/L glucose at 20 °C (**a**,**b**) and 38 °C (**c**,**d**).

**Figure 3:**
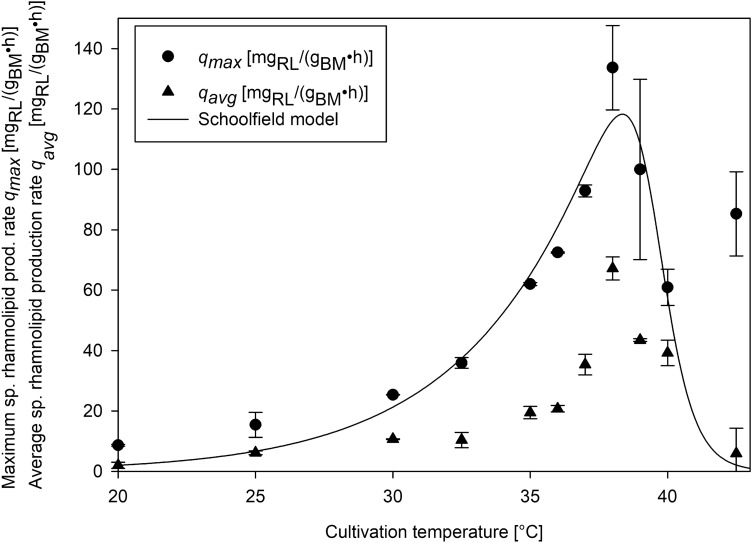
Experimentally determined maximum (*q*_*max*_; black circles) and mean (*q*_*avg*_; black triangles) specific rhamnolipid production rates plotted against temperatures tested for *Pseudomonas putida* KT2440 pSynPro8oT_4U_*rhlAB*. Maximum specific rhamnolipid production rates were described as a function of temperature by fitting a temperature model (Schoolfield et al.^30^; Eq. (1); black line) to the obtained data of *q*_*max*_.

When cultivating *P. putida* KT2440 pSynPro8oT_4U_*rhlAB* with the functional wild type fourU RNAT a lower biomass concentration was obtained at 38 °C (1.43 g/L), compared to 20 °C (5.71 g/L). However, the lower amount of biomass at 38 °C yielded a maximum concentration of RLs (1.84 g/L) approximately 10 times higher than the maximum RL concentration (0.19 g/L) measured at 20 °C. Compared to that the cultivation of the control strain with the destabilized fourU RNAT yielded 4.55 g/L biomass with 1.64 g/L RL at 20 °C and 0.49 g/L biomass with 0.48 g/L RL at 38 °C. At 42.5 °C almost no cell growth could be observed (0.10 g/L (inoculation)—maximum of 0.15 g/L (after 6 h)). In Fig. 2 selected time courses of shake flask cultivations of *P. putida* KT2440 pSynPro8oT_4U_*rhlAB* and *P. putida* KT2440 pSynPro8oT_4U(G14A-C25U)_*rhlAB* using ModR medium with 10 g/L glucose are shown. To visualize the effect of the different temperatures tested on the strains under investigation, maximum (*q*_*max*_) and mean (*q*_*avg*_) specific RL production rates were plotted against temperature (Fig. 3). Whereas *q*_*max*_ was chosen to illustrate and compare the highest rate recorded of each cultivation temperature tested and *q*_*avg*_ to display an average rate over the cultivation period for each cultivation temperature tested. A 15-fold increase of *q*_*max*_ from 9 mg/(g⋅h) at 20 °C to 133 mg/(g⋅h) at 38 °C was calculated. A model describing *q*_*max*_ as a function of temperature (Eq. (1)) according to the group of Schoolfield^30^ was fitted to the experimentally determined values of *q*_*max*_ for the *P. putida* strain harboring the functional fourU RNAT (Fig. 3). The experimentally determined values for *q*_*avg*_ respectively *q*_*max*_ increased from roughly 0 almost exponentially to a maximum of 67 mg/(g⋅h) (*q*_*avg*_) respectively 133 mg/(g⋅h) (*q*_*max*_) at 38 °C. The temperature at which the highest specific RL productivity could be observed (highest *q*_*max*_ from model) was calculated to be 38.4 °C when using the model of Schoolfield et al. At temperatures higher than 38 °C, experimentally determined RL production rates decreased rapidly. A mathematical artifact arising from a low biomass concentration at 42.5 °C, distorts the calculated value for *q*_*max*_ causing a high specific production rate at that temperature. Summarizing, the temperature responsive fourU element shows a pronounced thermo regulatory effect on RL production.

### Increased RL-per-glucose and RL-per-biomass yields at elevated temperatures

**Figure 4:**
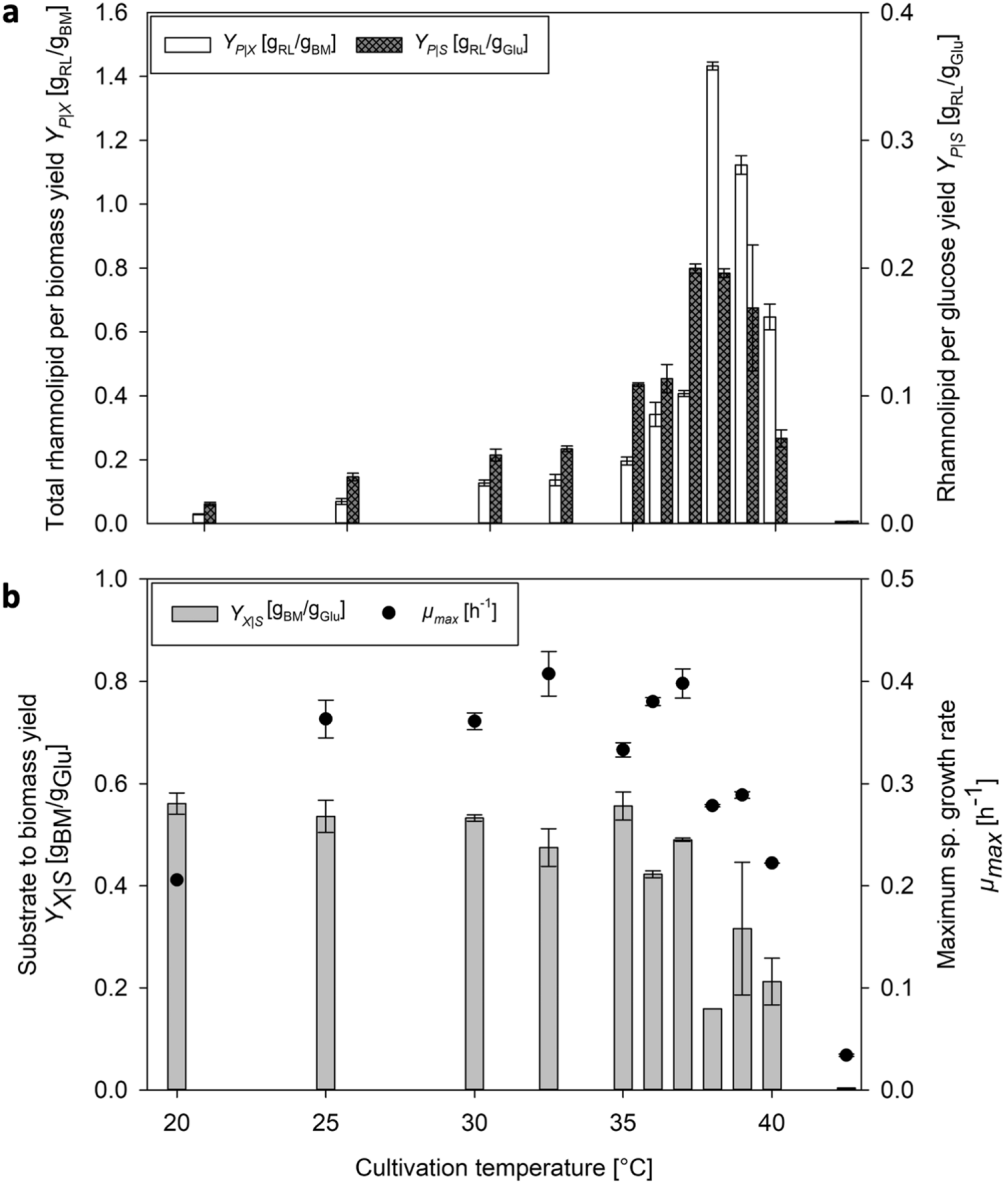
Effect of temperature on (**a**) rhamnolipid-per-glucose yields (*Y*_*P|S*_; dark grey bars) and total rhamnolipid-per-biomass yields (*Y*_*P|X*_; white bars) and on (**b**) biomass-per-glucose yields (*Y*_*X|S*_; grey bars) and maximum specific growth rates (*µ*_*max*_; circles) for *Pseudomonas putida* KT2440 pSynPro8oT_4U_*rhlAB.*

RL-per-glucose yields (*Y*_*P|S*_) and total RL-per-biomass yields (*Y*_*P|X*_) are shown for different temperatures for the functional fourU RNAT in Fig. 4a. An apparent correlation of *Y*_*P|S*_ respectively *Y*_*P|X*_, and temperature can be derived (Fig. 4a). The *Y*_*P|S*_ increased with the temperature by around 10-fold from 0.02 g/g (20 °C) to 0.20 g/g (38 °C). The *Y*_*P|X*_ value showed a similar trend with an even stronger increase when raising temperature. An almost 50-fold increase of the *Y*_*P|X*_ value between 20 °C and 38 °C from 0.03 g/g to 1.43 g/g was calculated. When temperature was raised higher than 38 °C both yield-values decreased approaching 0 at 42.5 °C. In addition to the performance parameters shown above, growth rates (*µ*) and biomass-to-glucose yields (*Y*_*X|S*_) were furthermore calculated (Fig. 4b). The highest maximum specific growth rates (*µ*_*max*_) were reached at 32.5 °C for the strain carrying the functional fourU RNAT with a *µ*_*max*_ of 0.41 h^-1^ respectively 0.33 h^-1^ at 35 °C for the control. *Y*_*X|S*_ remained relatively constant below and around the growth optimum (20–35 °C) with values around 0.50 g/g. Yields decreased at temperatures above 35 °C to 0.16 g/g at 38 °C before approaching almost 0 at 42.5 °C (Fig. 4b). As stated in the introduction, heterologous RL production suffers from considerable biomass production which represents a major carbon sink (roughly 50% more biomass than product)^14^. It is shown here that elevated temperatures, shift the RL-per-biomass yield in favor of RLs and simultaneously improve RL-per-glucose yields.

### Proof-of-concept temperature switch process

To benchmark the regulatory capabilities of the investigated fourU RNAT and to assess the production capacities of the applied biological system on a technical scale, a temperature switch process was carried out in a bioreactor. To achieve a separation of growth and product formation, cells were initially grown at 25 °C for biomass generation. At this temperature, RL formation rates were low, while biomass growth was high, as indicated in Figs. 3 + 4b. Subsequently, temperature was switched to 38 °C, which represents the experimental optimum of specific RL production rates. Switching the temperature from 25 to 38 °C was performed at t = 9 h, and a stable temperature could be reached within 15 min, with an error of less than 0.05 °C. The results are shown in the figure below. As expected, RL titers were lower than 0.10 g/L at 25 °C before induction and reached around 2.28 g/L after switching the temperature to 38 °C and 48 h of total cultivation time.

For the process shown above, maximum specific RL production rates of up to 145 mg/(g h) and average specific production rates of up to 32 mg/(g h) were reached. To put this simple batch process in context, a comparison was drawn with a literature high titer heterologous RL production process. The authors used the same strain, *P. putida* KT2440 pSynPro8oT_*rhlAB* without the fourU RNAT. Their cultivation was carried out in ModR medium with glucose as carbon source as well and at constant 30 °C (table below). The following table shows further reports on (heterologous) RL production processes using *Pseudomonas*.

**Figure 5:**
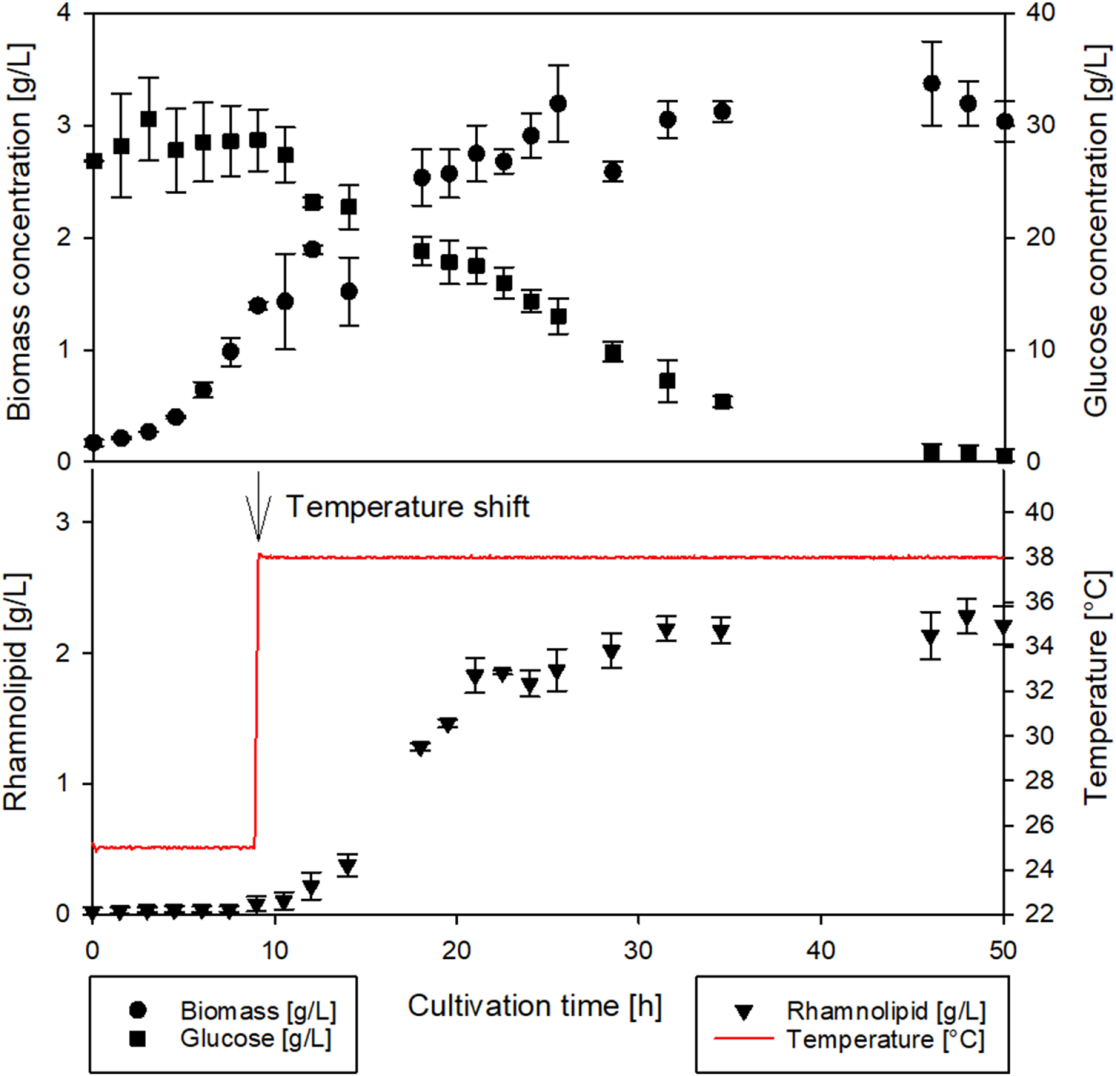
Bioreactor batch cultivation of *Pseudomonas putida* KT2440 pSynPro8oT_4U_*rhlAB* in ModR medium with 30 g/L glucose. Time courses of measured biomass- (circles), and glucose concentration (squares) are shown in the upper plot as well as rhamnolipid concentration (triangles) and measured temperature (red line) in the bottom plot. Biomass was grown at 25 °C until reaching an OD_600_ of 4 (approx. 1.4 g/L, after ~ 9 h). Then temperature was switched to 38 °C to induce rhamnolipid production as indicated by an arrow at the bottom plot.

The highest titer reported to date for mono-RL production, using a heterologous host, was reached in 2016 with a two phase feeding strategy at constant 30 °C^14^. For the switch process shown in this study average specific RL production rates (*q*_*avg*_) of up to 32 mg/(g h)) were reached. This result was around 25% higher compared to the highest average specific RL production rate (*q*_*avg*_: 24 mg/(g h)) reported in the literature reference process for heterologous RL production using *P. putida* KT2440 pSynpro8oT_*rhlAB*^14^ (Table 1; Fig. 5). This finding supports the potential of temperature to be exploited as control variable in combination with a functional RNAT. However, a suitable feeding strategy would still be required in conjunction with the RNAT-driven molecular control system to allow for higher titers and achieve reduced side product formation. The presented switch process in this study is a proof-of-concept for exploiting a temperature responsive RNAT for bioprocess control on a pre-industrial scale. To our knowledge the process is the first of its kind to leverage a fourU RNAT in combination with a temperature shift to control RL production.

**Table 1:** Comparison of rhamnolipid production processes from literature using glucose as carbon source with the presented switch process (Fig. 5).

Strain	*q*_*max*_ [mg_RL_/(g_BM_⋅h)]	*q*_*avg*_ [mg_RL_/(g_BM_⋅h)]	*Y*_*P|X*_ [g/g]	c_RL_^max^ [g/L]	References
*P. putida* KT2440 pSynPro8oT_4U_*rhlAB*	92–145	25–32	0.66–0.85	2.15–2.42	This study
*P. putida* KT2440 pSynPro8oT_*rhlAB*	72	42	0.61	0.83	^31^
*P. putida* KT2440 pSynPro8oT_*rhlAB*	89–113	18–24	0.66–0.77	14.9	^13, 14^
*P. putida* KT2440 SK4	N/A	N/A	0.35–0.37	0.68–0.74	^32^
*P. putida KT2440* E1.1_RL	N/A	N/A	0.41–0.43	1.01–1.03	
*P. aeruginosa* wt, various strains	N/A	12–54	0.3–2.3	0.2–39	^9, 33^

N/A = Information not available; c_RL_^max^ = Maximum rhamnolipid concentration.

## Discussion

**Figure 6:**
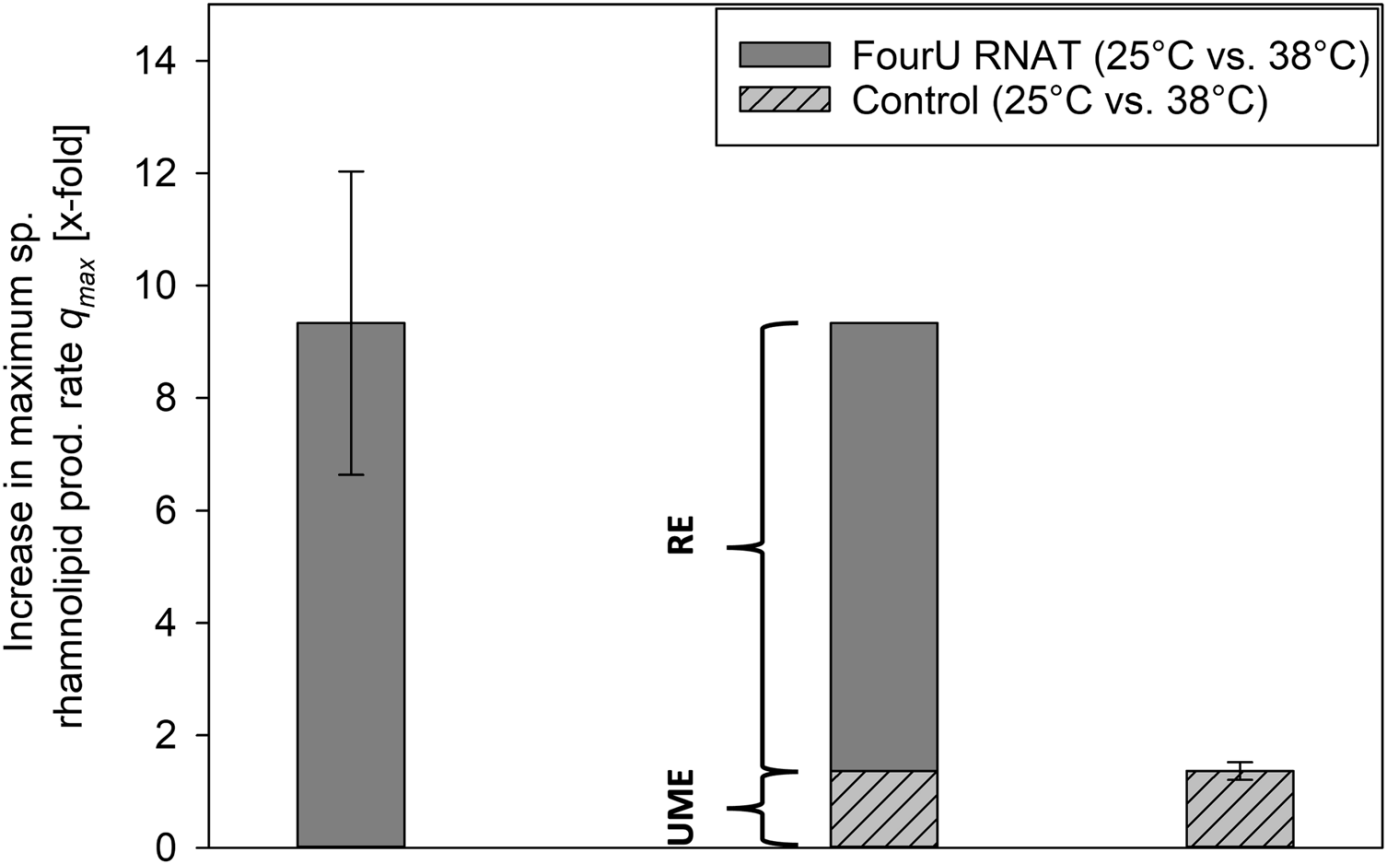
Comparison of increase in maximum specific rhamnolipid production rate (*q*_*max*_) of *Pseudomonas putida* KT2440 pSynPro8oT_4U_*rhlAB* and control strain when grown at 25 °C and 38 °C respectively. Average differences in production rate calculated from individual biological data are shown with derived regulatory effect (RE) and unspecific metabolic effect (UME).

When comparing time courses of biomass and RL concentration during shake flask cultivations of *P. putida* KT2440 pSynPro8oT_4U_*rhlAB* at 20 °C and 38 °C, the final biomass concentration at 38 °C was lower while the maximum RL concentration was almost 10-times higher than what was obtained at 20 °C (Fig. 2). Consequentially a higher biomass specific productivity was reached when cultivations were performed at elevated temperatures (Fig. 3). The temperature-dependency of RL production rates using fourU RNAT was investigated in a range between 20 and 42.5 °C (Fig. 3). An almost exponential increase of RL productivity between 20 °C to an optimum at 38 °C could be observed. In the absence of limitations and by assuming that RL production rate is proportional to rhamnosyl transferase concentration, this correlation mirrors what is known for RNAT regulation behavior. It has been reported before that the opening of the RNA hairpin structure exhibits a gradual temperature-response profile and typically never entirely prevents translation^34,35^. The rapid decrease in productivity when the optimal temperature for RL production is exceeded could be attributed to various effects of elevated temperatures on the bacterial cell. High temperatures are for example connected to aggregation and denaturation of proteins. This may lead to deactivation of enzymes needed for RL biosynthesis. Indirect effects arising from an altered bacterial metabolism may also be considered, such as the availability of precursors necessary for RL production. A lower availability of the RL precursors HAA and dTDP-rhamnose may then present a bottleneck for RL synthesis. Low temperature results in low growth which then leads to a reduced demand for dTDP because less DNA needs to be synthesized. This would mean that more dTDP nucleotide sugars (activated sugars) for RL biosynthesis may be available, thus resulting in higher RL production rates at the respective temperature. Furthermore, the substrate binding capacity of RhlA respectively RhlB towards the previously mentioned precursors may also be negatively affected by temperatures beyond the production optimum. The total productivity of the process may be evaluated according to the average specific RL production rate which can then ultimately be used as target for process optimization as has been suggested previously^25^. The observed increase of average specific RL production rate over temperature may differ in prolonged processes, for example in a bioreactor cultivation with a higher initial glucose concentration. For bulk chemical production, total *Y*_*P|X*_ respectively *Y*_*P|S*_ mark important parameters for process optimization and are therefore suitable targets to assess the production capacity of the applied biological system under control of the fourU RNAT. In this study, these yields were observed to be increasing alongside with increasing maximum respectively average RL production rates when raising temperature (Figs. 3 + 4). Translating this to the design of a more efficient production process at heightened temperatures would mean both a shortening of process time (increased RL production rates *q*) and higher efficiency (increased product per biomass respectively per substrate yields over temperature) of RL production. The higher efficiency of RL production at elevated temperatures may be assigned to different overlapping effects. To estimate the influence of a functional fourU RNAT on the observed increase of RL productivity over temperature, the G14A-C25U mutant was used as comparison. As has been reported previously, the RNA structure of the G14A-C25U mutant is destabilized already at a lower temperatures than the wild type RNA structure^29^. The increase of *q*_*max*_ and *q*_*avg*_ between 25 and 38 °C for the *P. putida* strain harboring the functional fourU RNAT was determined to be 9.3-fold respectively 11.1-fold compared to 1.4-fold for both rate changes of the G14A-C25U mutant (Fig. 3). Assuming on the one hand that the functional fourU RNAT has no significant regulatory effect on RL production rates, the increase of the rate over temperature for the G14A-C25U mutant should have been approximately the same. On the other hand, if an absence of unspecific or metabolic effects is assumed, the increase in production rate over temperature were to be solely caused by the RNATs regulatory effect. This would mean that the increase in production rate of the G14A-C25U mutant should have been close to 0. The here presented experiments revealed that besides the discussed metabolic effect, a major part of the total increase in RL production rate is to be attributed to the regulatory effect of the RNAT. The specific production rate between 25 and 38 °C for the G14A-C25U mutant increased 1.4-fold and may be due to unspecific metabolic effects (Fig. 3). Compared to that the specific production rate of the strain with the functional fourU RNAT increased by more than 9-fold on average between 25 and 38 °C, which confirms the regulatory action of the deployed fourU RNAT element in the heterologous host *P. putida* KT2440 (Figs. 3 + 6). The effect could be mainly assigned to the presence and regulatory effect of a functional fourU RNAT (RE, Fig. 6). The regulatory effect is derived by comparing specific productivities and time courses of cultivations with the strain carrying the functional fourU RNAT (e.g., 9.3-fold change of *q*_*max*_ comparing 25 to 38 °C, Fig. 6) with the control strain. In case of the control, only a slight increase in maximum specific productivity (e.g., 1.4-fold change of *q*_*max*_ comparing 25 to 38 °C, Fig. 6) was obtained. The observed increase in *q*_*max*_ for the control may be assigned to an unspecific metabolic effect (UME, Fig. 6) caused by the temperature increase itself along with elevated biochemical reaction rates. The UME accounts for 12 – 15% of the total change in average respectively maximum specific production rate over temperature as is shown below.

Different overlapping effects reported in connection to the RNATs, and their regulatory abilities have to be mentioned and considered. RNATs are often reported to have a high degree of “leakiness” respectively inefficiency when it comes to inhibition of translation^36^. Moreover, the regulatory potential of RNATs can only be harnessed by changing the temperature. In addition, temperature influences also the entire metabolism and individual metabolic conversion reaction rates can change by altered enzyme activity or even result in inactive enzymes due to denaturation of proteins at elevated temperatures^37^. This could consequently lead to an increase of substrate usage required in metabolic maintenance. An altered temperature also causes different levels of solubilized oxygen in the growth medium, with higher temperatures generally leading to a reduction in oxygen solubility. In this study, a plasmid with the *rhlAB* genes governed by the fourU RNAT was designed and used. The strong increase of RL production rates (> 9-fold) and yields (up to 10 respectively 50-fold for *Y*_*P|S*_ respectively *Y*_*P|X*_) with increasing temperature indicates a high potential for dynamic temperature adjustments to be used in process control during a RL production process. Figure 6 summarizes the effect of higher biomass specific productivity at elevated temperatures and elucidates its potential for a bioreactor switch process. In the batch reactor set-up, the applicability of the molecular RNAT combined with a switch in temperature (25 °C to 38 °C) for a RL production process (Fig. 5) was shown and compared to previous results for (high titer heterologous) RL production processes (Table 1). The initial temperature of 25 °C was chosen instead of 20 °C to ensure growth at a sufficiently high growth rate while still having low RL production rates (Figs. 3 + 4b) to separate growth and production phase. In this simple batch set-up incorporating a switch from 25 °C to 38 °C after reaching an OD_600_ of 4, the average specific RL production rate could be increased by up to 25% compared to the literature process^14^ (Table 1). As such, this batch process outlines the potential of using a RNAT based temperature switch for molecular bioprocess control. Furthermore, RNAT hold several advantages over conventional chemical induction systems such as no use of chemical inducers, (reversible) induction by temperature, possible autoinduction of the system by metabolic heat and earlier onset of the induction response (mRNA is already produced). In a large-scale industrial setup, that would potentially reduce overall costs for chemical inducers, enable tighter control of product-per-biomass ratios and allow for greater flexibility of process control. Furthermore, to track metabolic changes, future studies may also include transcriptome and metabolome analysis, which could point to bottlenecks in the bacterial metabolism and provide additional key factors for optimization approaches using metabolic engineering. Future investigations should aim for a gradual adjustment of temperature over time using tailor made temperature profiles along with an optimized feeding strategy to allow for higher product titers and reduce side product formation. Process modeling may be used to facilitate the completion of this challenging task, because temperature adjustments bring about a wide range of metabolic changes and an optimum temperature profile may therefore not be apparently conceivable. A suitable modeling tool to capture the complexity of a changing temperature during a bioprocess may be an artificial neural network. The combination of a trained artificial neural network and a genetic algorithm may be a powerful strategy to compute an optimized temperature profile to achieve high RL titers. By using temperature sensitive control elements in combination with a well-directed shift in temperature a key challenge in bioprocesses can be met. Substrate carbon can be more efficiently used by reducing by-product formation in form of biomass while substrate carbon is directed into the product. As such this step contributes to the development of sustainable and efficient bioprocesses for a future bioeconomy.

## Methods

### Chemicals and reference substances

The chemicals used in this study were purchased from Carl Roth GmbH (Karlsruhe, Germany) if not stated otherwise. The chemicals for analytic procedures were of analytical or mass spectrometry grade. Former Hoechst AG (Frankfurt-Hoechst, Germany) kindly provided the di-RL (Rha-Rha-C10-C10) standard used for HPTLC analysis. The mono-RL (Rha-C_10_-C_10_) standard was purchased from Sigma-Aldrich Laborchemikalien GmbH (Seelze, Germany). Derivatization of RLs was carried out as described previously^38^ using the derivatization agents 4-bromophenacylbromide and triethylamine which were obtained from Sigma-Aldrich Laborchemikalien GmbH (Seelze, Germany).

### Microorganism and plasmids

*Pseudomonas putida* KT2440 carrying the pSynPro8oT_*rhlAB* plasmid for heterologous production of mono-RLs was used as described previously^13^. The ROSE-like RNAT sequence on the pSynPro8oT_*rhlAB* plasmid as described previously^25^ was exchanged with either the functional fourU RNAT native to *Salmonella* or the destabilized sequence as control (Fig. 1)^29^. The sequences for the wild type fourU RNAT and the destabilized G14A-C25U mutant shown in Table 2 were ordered via Eurofins Genomics (Ebersberg, Germany) as a standard gene synthesis in a standard pEX A128 vector with ampicillin resistance for selection. Each fourU RNAT sequence variant ordered, was flanked by Flank A (Table 2) also present in the pSynPro8oT_*rhlAB* template plasmid containing the pSynPro8oT synthetic promotor as well as the *Bgl*II restriction site and downstream of the fourU RNAT sequence by Flank B (Table 2) coding for a part of the *rhlA* gene as well as the *Pci*I restriction site.

**Table 2:** Sequences used for construction of pSynpro8oT plasmid carrying a fourU RNA thermometer from *Salmonella* along with the control (G14A-C25U).

Name	Sequence (5′ → 3’)
FourU	GTTGAACTTTTGAATAGTGATTCAGGAGGTTAATG
G14A-C25U	GTTGAACTTTTAAATAGTGATTTAGGAGGTTAATG
Flank A; *BglII*	TCGCAGTCGGCCTATTGGTTAAGATCTTAACGCGCCAGCTCTTG ACAAGGTCGGAAAATTGAAGTATAATATCAGTCATCGGCTACGC GTGAACACGGACGCCAATCGTTTGCGCAGGCCGATCTGCAAGAC CCACACAAGCCCCTCGCCTGAAGGGGTACGCATCCGCCGTGGCT GGTCCGCGCGGATGGCCGCTGAGTT
Flank B; partial *rhlA*; *PciI*	ATGCGGCGCGAAAGTCTGTTGGTATCGGTTTGCAAGGGCCTGCG GGTACATGTCGAGCGCGTTGGGCAGGAT

The pEX plasmids containing the sequences described above were transferred into chemically competent *Escherichia coli* DH5α (New England Biolabs, Ipswich, USA) for plasmid propagation and isolation. The isolated pEX plasmids containing the different fourU RNAT variants were cut with *Bgl*II and *Pci*I. The pSynPro8oT_*rhlAB* backbone isolated from *P. putida KT2440* pSynPro8oT_*rhlAB* was cut analogously with *Bgl*II and *Pci*I to remove the ROSE-like RNAT and to produce matching sticky ends. After dephosphorylation of digested pSynPro8oT_*rhlAB* backbone and ligation with digested fourU RNAT fragment from pEX plasmid using T4 DNA ligase, the plasmids pSynPro8oT_4U_*rhlAB* and pSynPro8oT_4U(G14A-C25U)_*rhlAB* containing the different fourU RNAT variants instead of the ROSE-like RNAT were transformed into electrocompetent *Pseudomonas putida* KT2440 wild type cells. Electrocompetent *P. putida* KT2440 wild type cells were prepared according to^39^. Electroporation of electrocompetent cells was carried out with an electroporation device (Eporator, Eppendorf AG, Hamburg, Germany) and voltage set to 2.5 kV. Strains were selected on LB agar plates containing 20 mg/L tetracycline and confirmed with subsequent sequencing (Eurofins Genomics; Ebersberg, Germany). All strains were stored as glycerol stocks at -80 °C. The strains *Pseudomonas putida* KT2440 pSynPro8oT_4U_*rhlAB* and *Pseudomonas putida* KT2440 pSynPro8oT_4U(G14A-C25U)_*rhlAB* respectively are referred to as FourU and control in the following.

### Media preparation

All growth media (LB, SupM, ModR) were prepared as described previously^14^.

### Shake flask cultivation

Cultivations were performed in a shake incubator (New Brunswick/Innova 44, chamber, Eppendorf AG, Hamburg, Germany) using temperatures between 20 and 42.5 °C and 120 rpm. The first preculture was prepared in a 250 mL baffled shake flask, by inoculating 25 mL of LB medium containing 20 mg/L tetracycline with 50 µL from a glycerol stock solution of *P. putida* KT2440 pSynPro8oT_4U_*rhlAB* or *P. putida* KT2440 pSynPro8oT_4U(G14A-C25U)_*rhlAB* respectively. The first preculture was incubated at 120 rpm and 30 °C, grown for 24 h and subsequently 1 mL was used to inoculate 100 mL of SupM medium (seed culture) in 1 L baffled shake flasks as described by^14^. Each seed culture was grown for 15 h at 120 rpm and 30 °C and used to inoculate the main culture in ModR medium to an OD_600_ of 0.3 respectively 0.5 for bioreactor cultivations.

### Bioreactor cultivation

The bioreactor cultivation using a 2 L tabletop bioreactor (Minifors 4, Infors HT, Bottmingen, Switzerland) was carried out as described previously^14^ with minor changes. The bioreactor cultivation was carried out in duplicates and as a batch cultivation using 30 g/L glucose. Temperature was switched from 25 °C to 38 °C when reaching an OD_600_ of around 4 after approximately 9 h of cultivation. The antifoam SB590 (Schill + Seilacher, Hamburg, Germany) was added if needed.

### Sample processing

Sample processing was carried out as previously described^14^. To extract RLs, culture supernatant was first acidified 1:100 (*v*/*v*) using 85% phosphoric acid. Subsequently extracted two times with 1.25:1 (*v*/*v*) ethyl acetate, then pooled and evaporated in a vacuum centrifuge at 40 °C for 40 min and 10 mbar (RVC 2-25 Cdplus, Martin Christ Gefriertrocknungsanlagen GmbH, Osterode am Harz, Germany).

### Quantification of RLs with HPTLC protocol

Detection of RLs was carried out as described previously^38^ with a modified protocol for HPTLC^40^. Briefly, RL samples were re-dissolved in acetonitrile after evaporation and derivatized using a 1:1 (*v/v*) mixture of 135 mM bromphenacylbromid and 67.5 mM tri-ethyl-ammonium/-amin. The derivatization step was performed for 90 min at 1400 rpm and 60 °C as previously described^41^. Expected RL concentrations exceeding 2 g/L were diluted prior to derivatization. In a next step, derivatized samples were measured on silica gel 60 HPTLC plates with fluorescence marker F^254^ (Merck, Darmstadt, Germany). Measurements were carried out on a HPTLC system for quantitative analysis (CAMAG Chemie-Erzeugnisse & Adsorptionstechnik AG, Muttenz, Switzerland). Samples application was performed with the Automatic TLC Sampler 4 (ATS 4). Plate development was carried out with the Automatic Developing Chamber 2 (ADC 2), which is equipped with a 20 cm × 10 cm twin-trough chamber. Developed plates were subsequently analyzed with the TLC Scanner 4. A HPTLC imaging and data analysis software (winCATS 1.4.7.2018 software, CAMAG, Muttenz, Switzerland) was used to control the HPTLC-system. Filling speed of the syringe for sample application was set to 15 µL/s and dosage speed to 150 nL/s. The syringe was rinsed with methanol in between sample application. Application start was at 15 mm from the left and 8 mm from the lower edge and band width set to 6 mm. For plate development a mobile phase composed of 30:5:2.5:1 isopropyl acetate:ethanol:water:acetic acid was used. Tank saturation was set to 5 min and subsequent drying was carried out for 5 min with a stream of air. For scanning a deuterium (D2) lamp with slit dimensions of 3 mm × 0.3 mm was used. Plates were scanned at 263 nm at which wavelength bromphenacyl-derivatized RL congeners absorb. For data resolution and scanning speed 1 nm/step and 100 nm/s respectively were chosen.

### Enzymatic assay

For quantification of glucose content in the culture supernatant of samples, an enzymatic glucose assay kit (R-Biopharm AG, Darmstadt, Germany) was used, according to the manufacturers’ instructions and scaled down to 96-well format.

### Graphical analysis, regression and replicates

The scientific graphing and data analysis software (SigmaPlot, Systat Software Inc., San Jose, CA) was used to carry out regression analysis of measured data. A four-parameter logistic fit for biomass, glucose and RL concentration, was used to illustrate the time course of these variables^42^. Measurement results and calculated performance parameters (rates and yields) are presented as mean ± standard deviation obtained from data of duplicates from at least two independent biological experiments. To avoid mathematical artifacts caused by low biomass concentrations, specific RL production rates (*q*) were calculated starting at hour 3.

### Parameter optimization, modeling and simulation

MATLAB 2020b (The MathWorks, Natick, MA, USA) was used to perform parameter fitting and simulation for the temperature-dependency model, shown in the results section. The Nelder-Mead numerical algorithm implemented in the embedded function “fminsearch” was used for parameter optimization. Optimization using the functions described above was performed by minimizing the error of simulation data and measured data according to a least-square error function. To demonstrate and describe temperature dependency of specific RL production rates, a mechanistic temperature model (Eq. (1)) was used for a fitting curve^30^.

1 $$q_{max} \left( T \right) = \frac{{\mu_{25} \cdot \frac{T}{298} \cdot e^{{\left[ {\frac{{H_{a} }}{R} \cdot \left( {\frac{1}{298} - \frac{1}{T}} \right)} \right]}} }}{{1 + e^{{\left[ {\frac{{H_{l} }}{R} \cdot \left( {\frac{1}{{T_{l} }} - \frac{1}{T}} \right)} \right]}} + e^{{\left[ {\frac{{H_{h} }}{R} \cdot \left( {\frac{1}{{T_{h} }} - \frac{1}{T}} \right)} \right]}} }}$$

## Data Availability

All obtained data has been included into the manuscript. Please turn to the corresponding author for all other requests.
